# Characterization of Crater Area in a Target Penetrated by a Wf/Zr-Based Amorphous Matrix Composite Projectile

**DOI:** 10.3390/ma13235523

**Published:** 2020-12-03

**Authors:** Xianghai Ye, Minming Zou, Jiankang Chen

**Affiliations:** 1 School of Mechanical Engineering and Mechanics, Ningbo University, Ningbo 315211, Zhejiang, China; xianghaiye@yeah.net; 2 Inner Modern Mold College, Zhejiang Vocational and Technical College of Industry and Commerce, Ningbo 315012, Zhejiang, China; zmmchp@126.com

**Keywords:** Wf/Zr-based amorphous matrix composite, adiabatic shear band, penetration, 4340 steel

## Abstract

Tungsten fiber-reinforced Zr_41.25_Ti_13.75_Cu_12.5_Ni_10_Be_22.5_ amorphous matrix composites (hereinafter referred to as Wf/Zr-based amorphous matrix composites) are considered as a potential new generation of projectile material, while the penetration behavior of Wf/Zr-based amorphous matrix composites is not fully clear yet. In order to better understand the penetration behavior of this composite material and study its armor-piercing performance, a ballistic experiment was performed and the hardness and microstructure around the crater of a target material were studied. A ballistic experiment was performed with a projectile of Wf/Zr-based amorphous matrix composite and a target of 4043 steel. After the ballistic experiment, the target was cut through the crater using a wire cutting machine into a sample with size 150 mm × 40 mm × 20 mm, which was later polished by different types of sandpaper. The micro-hardness was analyzed in a micro-hardness tester, and the microstructure was observed by SEM. According to this study, three layers were identified in the direction lateral to the crater, consisting of a martensite layer, a deformation strengthening layer, and the original structure layer. Moreover, the martensite layer initially thickened and then thinned in the direction longitudinal to the crater.

## 1. Introduction

There is an urgent need to improve the armor-piercing performance of projectiles since the armor-piercing resistance of target materials has increased in recent years [1,2]. Projectile materials are used to make projectile in penetrator, and the armor-piercing performance of the penetrator is mainly determined by the kind of projectile material. Projectile materials consist mainly of tungsten alloy and depleted uranium alloy [3,4]. Tungsten alloy mainly contains W, Ni and other alloying elements. Due to the heavy density of tungsten, tungsten alloy can penetrate armor steel. While during the penetration of a tungsten alloy projectile, the alloy grains can be bent and extremely deformed opposite to penetration direction [4], causing the volume in front of the projectile to be larger than that in the back, and this phenomenon, called mushroom head, decreases the armor-piercing performance of the projectile. Depleted uranium alloy contains U238, U235 and other alloy elements. Because of the heavy density of uranium, depleted uranium alloy can also penetrate armor steel easily. As shear bands which are 45° with the penetration direction are formed in penetration of depleted uranium alloy, the volume in front of the projectile is smaller than that in the back, and this phenomenon called self-sharping character increases the armor-piercing performance of the projectile. The armor-piercing property of depleted uranium is better than that of tungsten alloy, while the depleted uranium alloy is unfriendly to the environment because of radioactivity and chemical toxicity. Therefore, it is urgent to develop a new kind of projectile material which has both outstanding armor-piercing performance and pollution-free property.

Wf/Zr-based amorphous matrix composites were first prepared by Dandliker et al. in 1998 [5]. Afterward, the mechanical and dynamic properties of Wf/Zr-based amorphous matrix composites were further studied by Dandliker et al., and it was found that its penetration performance was 10–20% better than that of tungsten alloy [6,7], which demonstrated that this composite can be used as a novel projectile material instead of depleted uranium alloy. Thereafter, studies regarding this material increased worldwide [8,9]. In the following decades, studies have focused on the fracture mode, numerical simulations, and self-sharpening characteristics of the composites, and important achievements have been made [10,11,12].

However, there is still a need to study the penetration behavior and mechanics of Wf/Zr-based amorphous matrix composites, which will help understand the projectile and improve its penetration performance. More specifically, the observation of the microstructure of the target material or residual projectile is a common and effective method to study its penetration behavior. Several achievements have been made by using this method to study the penetration behavior of tungsten alloy and depleted uranium alloy projectiles. For example, adiabatic shear bands (ASB) have been found in depleted uranium alloys which served as a self-sharpening mechanism, and ASB were also discovered in Wf/Zr-based amorphous matrix composite fragments [3]. The discovery of ASB is beneficial to understanding projectile behavior and mechanism. ASB are very common under high strain conditions, and they have been observed during the deformation of a series of materials, such as metal, ceramics, and metal matrix composites [13,14,15,16,17]. ASB are formed during thermal softening because adiabatic heating exceeds strain hardening [18]. By observing the microstructure of the target or residual projectile, Herve Couque showed the negative correlation of the critical equivalent plastic strain at the shear band initiation sites and the penetration performance of tungsten alloy [19]. Additionally, Dong-Kuk Kim found that the width of adiabatic shear bands in remaining tungsten alloy projectiles was wider than that of the shear band in armor plates [20]. Moreover, Uwe Gerlach explained the penetration mechanism of the heavy tungsten alloy by microstructural analysis of residual projectiles [4]. Last, Z. Q. Duan studied the performance of tungsten alloys against two different targets by observing the microstructure of the target around the crater [21].

In this study, a ballistic experiment was conducted and the 4340 steel was selected as a target, because 4340 steel is generally used to evaluate the penetration performance of new kind of projectiles. As mentioned above, to study the penetration behavior of projectile more deeply, the methods of hardness analysis and microstructure observation of the target around the crater were taken.

## 2. Materials and Methods

**Figure 1:**
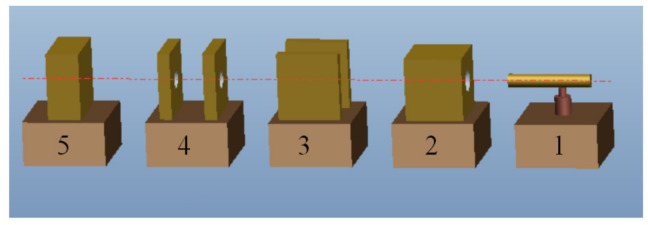
Schematics of the ballistic experiment: 1 is a 30 mm ballistic gun, 2 is the sabots collector, 3 is a high-speed camera, 4 is a speed measurement system, 5 is the target.

A ballistic experiment was performed with a projectile and target material consisting of Wf/Zr-based amorphous matrix composite and 4043 steel, respectively. The sizes of the projectile and target were ∅ 6 mm × 93 mm and 300 mm × 200 mm × 150 mm (length × width × thickness), respectively. The material information of the projectile and target is given in Table 1, and the schematics of the experiment are illustrated in Figure 1.

**Figure 2:**
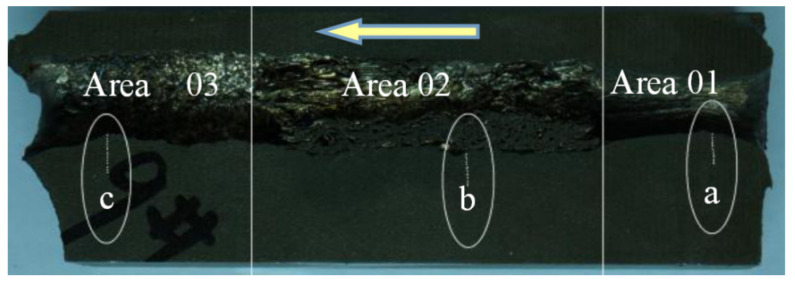
Picture of the target sample.

In this experiment, the speed of the projectile was determined by the weight of the gunpowder, using the high-speed camera and speed measurement system (XGK-2002, Xi’an Technological University, Xi’an, China). The speed of projectile was 1200 m/s, and the angle between trajectory and target was 90°. After the ballistic experiment, the target was cut through the crater using a wire cutting machine into a sample with size 150 mm × 40 mm × 20 mm (length × width × thickness), which was later polished by different types of sandpaper. A typical target sample is shown in Figure 2. The arrow shows the ballistic direction, and the target sample can be divided into 3 areas (01, 02, and 03) which show different hardness distributions. The elliptical regions a, b, and c in Figure 2 are the SEM observation areas, and the lines in the ellipses describe the spots of hardness measurements. The micro-hardness was analyzed in a micro-hardness tester (MH-50D, Hengyi Company, Shanghai, China), and the microstructure was observed by SEM (QUANTA FEG250, FEI Company, Hillsboro, OR, USA; SU-70, Hitachi Company, Tokyo, Japan). The load was 300 gf (2.942 N), and the duration time was 10 s in micro-hardness test in this paper.

## 3. Results and Discussion

### 3.1. Results

**Figure 3:**
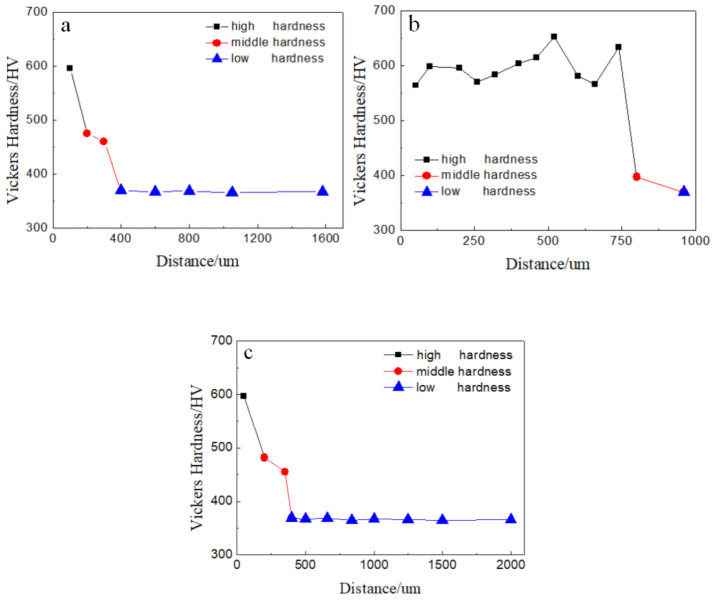
Hardness value as a function of distance from the edge of the crater. (**a**) Region a; (**b**) Region b; (**c**) Region c.

During the ballistic experiment, the projectile passed through the target. After the experiment, a target sample was prepared as shown in Figure 2. The three a, b, and c regions of the target around the crater shown in Figure 2 were tested using a micro-hardness tester, and the hardness results are presented in Figure 3. The *X* axis in Figure 3 corresponds to the distance between the test point and the edge of the crater, while the *Y* axis is the hardness of the test point.

**Figure 4:**
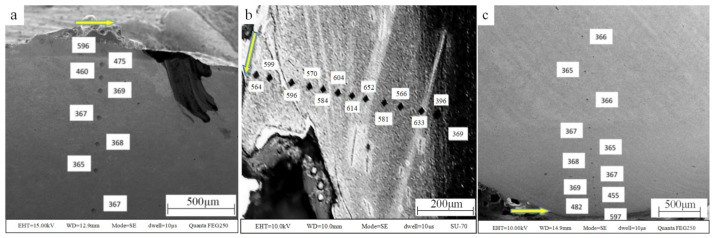
Microstructure and test point of target around crater: (**a**) Region a; (**b**) Region b; (**c**) Region c.

The target around the test points were observed by SEM, and the microstructure of the target is presented in Figure 4, where the hardness value was marked next to the test point, and the arrow represents the penetration direction. As is shown in Figure 4, adiabatic shear bands appeared in the test region b, and the hardness value of the adiabatic shear band was higher than that of the target outside the adiabatic shear bands.

By observing the morphology and testing the hardness along the crater, it was found that the three test regions of Figure 3a–c can represent the three areas of Figure 2 01, 02, 03. Three hardness ranges can be observed in Figure 3, which are the high hardness range (>500 HV), the middle hardness range (390–500 HV), and the low hardness range (<390 HV). It can be observed that the hardness increased as the distance between the test point and the edge of the crater decreased. More high hardness test points were detected in Region b compared with Regions a and c. In other words, the area of high hardness tested in Region b was larger than that of Regions a and c.

### 3.2. Discussion

**Figure 5:**
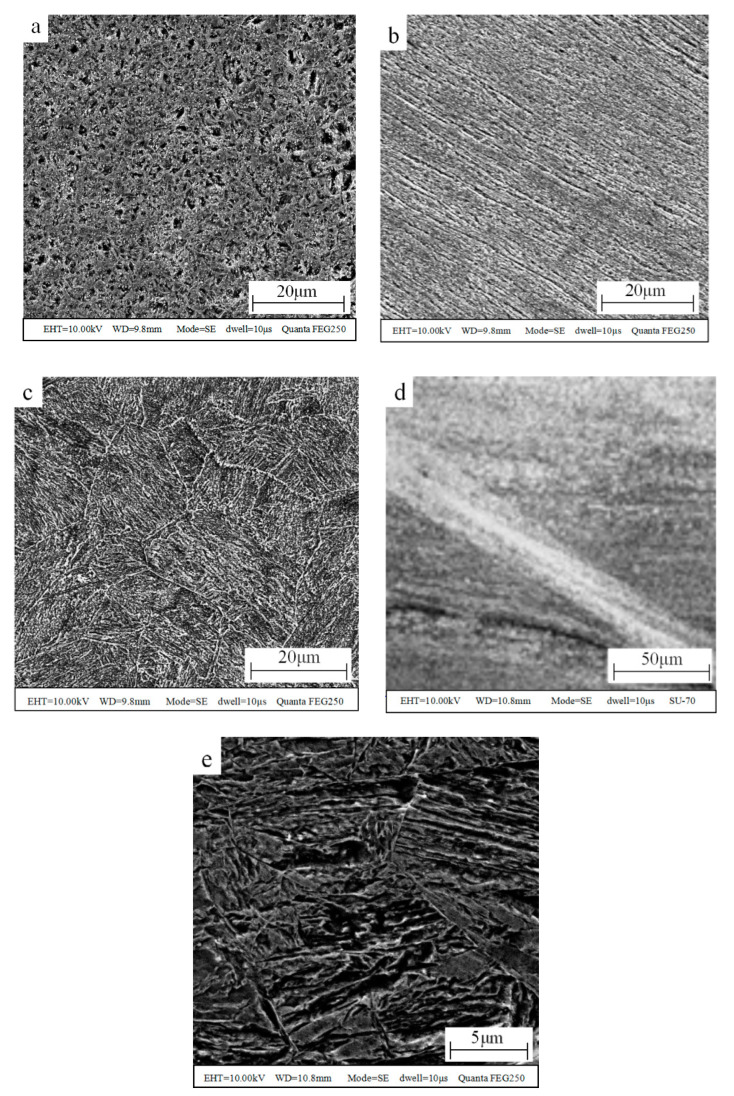
Microstructure images at higher magnification. (**a**) high hardness target, (**b**) middle hardness target, (**c**) low hardness target, (**d**) adiabatic shear band, (**e**) enlarged view of high hardness target.

In order to understand the reasons for the above phenomenon, an image of the microstructure at a higher magnification is shown in Figure 5, in which (a) is the typical microstructure image of high hardness target, (b) is the typical microstructure image of the middle hardness target, (c) is the typical microstructure image of the low hardness target, (d) is the microstructure image of the adiabatic shear band, and (e) is an enlarged view of (a).

As is shown in Figure 5, it was found that the microstructure in Figure 5a,e shows the typical feature of lath martensite, the microstructure in Figure 5b shows plastic deformation texture, the microstructure in Figure 5c presents the typical morphology of sorbite, which is the original structure of the target, and the microstructure in Figure 5d presents the typical morphology of adiabatic shear bands. It was shown that the grain size in Figure 5c is similar to that in Figure 5e. From Figure 5, considering the hardness, grain size and microstructure, it can be determined that the target material in the high hardness region (>500 HV) becomes martensite, while the target material in the middle hardness region (390–500 HV) was strengthened by deformation. Additionally, the target material in the low hardness region (<390 HV) is that of the original structure. There are two reasons for the oscillation distribution in Figure 3b: one is that the distance between the detection points is close and not fixed, and when the distance between the two indentations is less than three times the diagonal, the detection result will be higher than the actual situation to a certain extent because of the deformation strengthening; the other is that adiabatic shear bands appeared, and the hardness in the adiabatic shear bands was higher than that outside the adiabatic shear bands due to fine grain strengthening [22]. The test sequence was from the crater surface to the inner of the target. By carefully observing the distribution of test points, the test result of the second test point (599 HV) was only affected by the distance, the test result of the third test point (596 HV) was not affected by the distance or the adiabatic shear bands, the test results of the fourth to tenth test points were affected by the distance and the adiabatic shear bands, and the test result of the eleventh test point (633 HV) was only affected by the adiabatic shear band. It was found that the effect of adiabatic shear bands was greater than that of the distance. As mentioned above, adiabatic shear bands can appear in penetration when the thermal softening of adiabatic heating exceeds strain hardening. Adiabatic shear bands can be regarded as energy loss routes, and it was found that for Area 02 this is the main area of energy loss during penetration.

Overall, the formation of three different layers has been identified in the target around the crater, which are a martensite layer, a deformation strengthening layer, and the original structure layer. This phenomenon is caused by high-temperature and high-pressure conditions. As is known, the temperature during penetration can reach several thousand degrees centigrade and the pressure can reach dozens of Giga Pascal. The target material far away from the crater is insignificantly influenced by the high temperature and pressure, resulting in the material remaining in its original structure. At more moderate distances between the target material and the crater, the effect of high pressure exceeded that of high temperature due to the non-direct contact between the crater and the target material, and the material becomes a deformation strengthening layer. As the distance from the crater decreased, the effect of high temperature exceeded that of high pressure because deformation can be concealed in high temperature, and the material becomes a martensite layer.

The formation of martensite requires both austenitizing temperature and a sufficient cooling rate, and both of them are satisfied in the experiment. First, the original structure of 4340 steel is sorbite, and sorbite can be transformed into austenite when the temperature reaches the austenitizing temperature. Previous reports have shown that the temperature caused by penetration is very high, even exceeding the melting point of the target material [10,11], so the austenitizing temperature is satisfied. Second, the target with a size of 300 mm × 200 mm × 150 mm is large enough compared with the projectile, with excellent thermal conductivity, so the high temperature can be cooled instantly since the cooling rate was higher than the critical cooling rate of martensite transformation.

In order to verify that the temperature of the target near the crater exceeded the austenite temperature, the temperature in the adiabatic shear bands was calculated theoretically, considering that the main deformation type in penetration is plastic deformation. Previous studies showed that the heat energy increasing the temperature during penetration is about 90% of the plastic deformation energy [23], so the Marc Andre Meyers equation can be used to calculate the increase in temperature ($\Delta T_{P}$) of the target in the shear bands as follows [24]:

(1) $$\Delta T_{P}=\frac{0.9}{\rho C_{p}}\int_{0}^{\varepsilon_{f}}\sigma d\varepsilon$$

in which ρ is the density of the target material (4340 steel), *C*_p_ is the isobaric heat capacity of the target material, and σ and ε are the stress and strain of the target material, respectively. The relationship between stress and strain can be described by the Johnson–Cook model:

(2) $$\sigma =(A+B\varepsilon^{n})(1+C\ln\frac{\overset{•}{\varepsilon }}{{\overset{•}{\varepsilon }}_{0}})[1-{\left(T^{*}\right)}^{m}]$$

**Figure 6:**
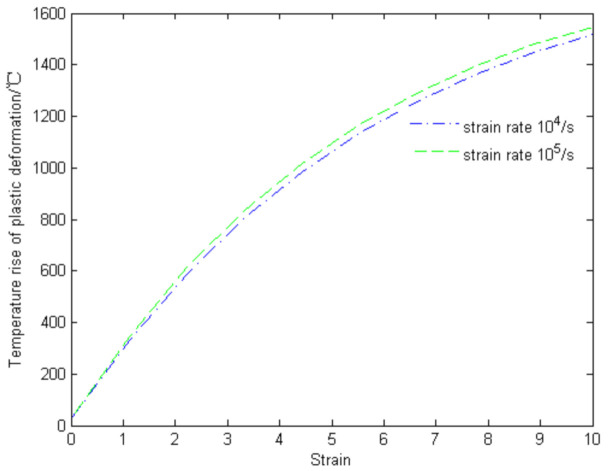
Theoretical calculation results of temperature in adiabatic shear bands.

The Johnson–Cook model parameters of 4340 steel are listed in Table 2 [25]. The temperature increase in the shear bands can be obtained by substituting the Johnson–Cook model into Equation (1). Figure 6 illustrates the results of the theoretical calculations, which are in good agreement with those in the literature [26]. The results confirmed that the temperature of the target in the ballistic experiment reached the austenization temperature, providing the necessary conditions for the formation of martensite.

Additionally, it should be highlighted that the appearance of martensite indicates that high temperature is produced. This martensite region is soft in the process of penetration, which is favorable for armor piercing. From the previous analysis, it is shown that a large area of original 4043 steel which transforms into martensite is softened in penetration, the armor-piercing performance of Wf/Zr-based amorphous matrix composite in the experiment is strong, and Wf/Zr-based amorphous matrix composite can be used as a new kind of projectile material.

After discussing the formation of the three layers and the mechanism of martensite transformation above, the distribution of martensite along the crater is discussed as follows.

More specifically, the martensite region is the austenite region in the process of penetration, so it is influenced by temperature and heating time.

On the one hand, as mentioned above, 90% of the deformation energy is converted into heat energy which causes the temperature rise. It is known that deformation energy depends on the compression degree or the force between target and projectile. In other words, the temperature in the target is affected by the compression or the force between the target and the projectile. The force between the target and the projectile decreased during penetration [27], so the temperature increase caused by compression during penetration decreased along the crater.

On the other hand, as the speed of the projectile decreased during penetration, the heating time for the same length of the target increased.

Combining the temperature and heating time parameters, the phenomenon of martensite layer distribution along the crater can be easily understood. Moreover, penetration can be divided into three stages, which are the initial launching stage, the steady penetration stage, and the shear plugging stage [27]. In the initial launching stage, the temperature increase caused by compression was the highest, while the heating time was the shortest, so the austenization region was small. As a result, the martensite layer formed in Area 01 was thin. In the steady penetration stage, the temperature and the heating time were both enough to form the austenization region, so the martensite layer formed in Area 02 was thick. In the shear plugging stage, the temperature increase caused by compression was the lowest, while the heating time was the longest, so the austenization region was also small. As a result, the martensite layer formed in Area 03 was thin. Therefore, the martensite layer (>500 HV) thickened first and then thinned in the longitudinal direction of the crater.

## 4. Conclusions

- It was found that three layers were formed in the lateral direction of the crater, namely, the martensite layer, the deformation strengthening layer, and the original structure layer. Moreover, the martensite layer thickened first and then thinned in the longitudinal direction of the crater.
- ASB occurred in the martensite layer in Area 02, thus leading to an oscillation distribution of the hardness of the target around the crater. The theoretical temperature in ASB can reach up to 1520 °C when the strain rate is 10^5^ and the strain is 10, and the high temperature transfers to the target steel around the ASB, which turns into the martensite layer.
- It was proved that the heat and high temperature were generated by the formation of ASB and the martensite layer, and the performance of target steel decreased since it is softened in such a high-temperature range. This phenomenon benefits the armor-piercing performance of Wf/Zr-based amorphous matrix composite projectiles. The armor-piercing performance was shown to be strong in the experiment, indicating that Wf/Zr-based amorphous matrix composite can be used as a new kind of projectile material.

## Figures and Tables

**Table 1:** Material information of projectile and target.

Condition	Chemical Composition	Original Structure of Material	Characteristic Temperature
Projectile	80% W 20% Zr_41.25_Ti_13.75_Cu_12.5_Ni_10_Be_22.5_ (Volume fraction)	Crystal structure of tungsten fiber + amorphous matrix	Melt temperature of W: 3683 K Melt temperature of Zr_41.25_Ti_13.75_Cu_12.5_Ni_10_Be_22.5_: 993 K
Target	0.37–0.44% C, 0.17–0.37% Si, 0.5–0.8% Mn, 0.6–0.9% Cr, 0.15–0.25% Mo, 0.5–0.8% W, ≤0.3% S, 0.25–1.65% Ni (Weight fraction)	Sorbite	Melt temperature: 1793 K

**Table 2:** Johnson–Cook model parameters of 4340 steel.

*A* (MPa)	*B* (MPa)	*n*	*C*	*m*
792	510	0.26	0.0147	1.03
